# Follicular Lymphoma Presenting as a Primary Omental Mass: A Case Report and Pathological Analysis

**DOI:** 10.7759/cureus.73810

**Published:** 2024-11-16

**Authors:** Srinivasan Raman, Kalaivani Amitkumar, Balaji Radhakrishnan, Subhalakshmi Kumaran

**Affiliations:** 1 Department of Pathology, Sri Ramaswamy Memorial (SRM) Medical College Hospital and Research Center, Sri Ramaswamy Memorial Institute of Science and Technology (SRMIST), Chengalpattu, IND; 2 Department of Radiology, Sri Ramaswamy Memorial (SRM) Medical College Hospital and Research Center, Sri Ramaswamy Memorial Institute of Science and Technology (SRMIST), Chengalpattu, IND

**Keywords:** extranodal lymphoma, follicular lymphoma, incidental finding of lymphoma, lymphoma, lymphoproliferative disorder, non-hodgkin lymphoma, primary mesenteric neoplasm, primary omental lymphoma, primary omental mass, rare lymphoma presentation

## Abstract

Follicular lymphoma (FL) is a gradually progressing type of B-cell non-Hodgkin lymphoma (NHL), distinguished by its characteristic follicular pattern of growth and a typically indolent clinical course. It is identified by the abnormal growth of B-cells in the lymph nodes. We report a case of a 45-year-old female who came up with complaints of heavy menstrual bleeding and easy fatiguability. Imaging studies revealed an accidental finding of a primary mesenteric mass. Infracolic omentectomy and a bilateral pelvic lymph node dissection were done, and the specimen was sent for histopathological examination. Microscopic examination of the omental mass revealed a nodular growth pattern with prominent follicular structures composed of centrocytes and scattered centroblasts. Immunohistochemical staining demonstrated positivity for cluster of differentiation 20 (CD20), CD10, and B-cell lymphoma 2 (BCL2), confirming the diagnosis of FL. Thus, we report this rare manifestation of FL presenting as an isolated omental mass.

## Introduction

Follicular lymphoma (FL) is classified as a subtype of non-Hodgkin lymphoma (NHL) distinguished by the growth of B cells that have a morphology similar to follicular lymphoid structures. FL cells originate from germinal center B-cells within the lymphoid follicle [1]. It is a disease that mainly impacts older adults, typically around 55 years of age, and is relatively infrequent in children [2].

Lymphoma is categorized into two groups: Hodgkin lymphoma (HL) and NHL, with NHL accounting for about 90% of cases [2]. NHL is a type of cancer that affects mainly the lymph nodes. It arises from B cell precursors, mature B cells, T cell precursors, and mature T cells [3]. The most prevalent types of neoplasms in mature B cells include diffuse large B cell lymphoma (DLBCL) and FL, followed by mantle cell lymphoma (MCL) and marginal zone lymphoma (MZL) [3]. In India, FL constitutes 7.2% of all NHL cases, reflecting a lower incidence compared to Western populations [4].

According to the recent World Health Organization (WHO) classification, frequent extranodal locations include the bone marrow, spleen, liver, and peripheral blood [5]. FL usually involves lymph nodes, particularly cervical and inguinal, but can also affect central nodes in the abdomen and chest [6]. NHL may spread through the lymphatic system or appear as an extranodal disease [3], with the gastrointestinal (GI) tract being the most commonly affected extranodal site, accounting for 30-40% of extranodal lymphomas [7,8] and 4-20% of all NHL cases [9,10]. Studies show that extranodal lymphomas commonly affect the GI tract, skin, bone, and brain [11-12]. Primary mesenteric tumors are rare, occurring in fewer than 1 in 200,000 cases, with FL being the most prevalent type [13,14]. Lymphoma rarely affects the omentum, a peritoneal fold made of fibrofatty tissue lacking lymphoid tissue [15]. We present a case of FL that presented at an unusual site from our institution.

## Case presentation

This case features a 45-year-old woman who presented with symptoms of excessive menstrual bleeding for three months, accompanied by easy fatigability and expulsion of blood clots. Her medical history includes a recent diagnosis of diabetes mellitus. She has no known history of systemic hypertension, bronchial asthma, tuberculosis, or thyroid disorders. Notably, there is no history of prior hospitalizations or blood transfusions.

**Figure 1:**
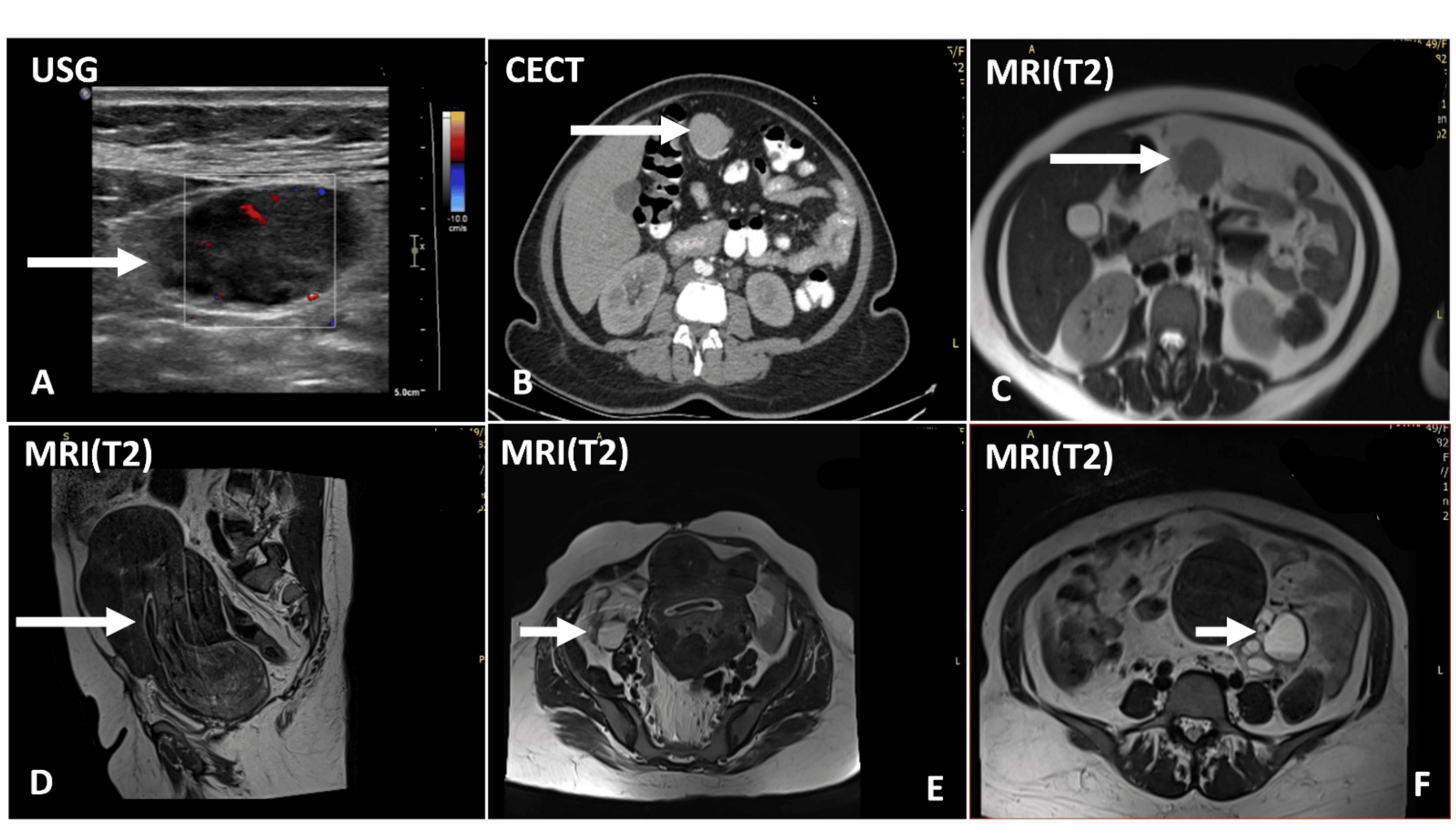
USG of the abdomen (A) shows a relatively well-defined hypoechoic lesion with areas of internal vascularity in the epigastric region (box in A). CECT of the abdomen (B) shows a homogeneously enhancing soft tissue density lesion in the epigastric region (arrow in B). MRI of the abdomen (C) shows a T2 isointense lesion in the epigastric region (arrow in C), a large submucosal fibroid (arrow in D), a right ovarian cyst (arrow in E), and a left ovarian cyst (arrow in F) USG: ultrasonogram; CECT: contrast enhanced computed tomography; MRI: magnetic resonance imaging

Imaging studies were conducted to further investigate her symptoms. Ultrasonography (USG) of the whole abdomen revealed a relatively well-defined hypoechoic lesion noted in the epigastric region with areas of internal vascularity (Figure 1A). Computed tomography (CT) of the abdomen revealed a well-defined, homogenously enhancing soft tissue density lesion in the mesentery (Figure 1B), raising the possibility of either a lymph nodal mass or a primary mesenteric neoplasm, with a recommendation for histopathological (HPE) correlation. Subsequently, magnetic resonance imaging (MRI) of the abdomen and pelvis was done, which confirmed a solid lesion in the epigastric region of mesentery with a few adjacent enlarged nodes (Figure 1C), suggesting possible secondary nodal deposits or a primary mesenteric neoplasm, warranting HPE correlation. Other findings included a bulky uterus with multiple large fibroids, including a large submucosal fibroid prolapsing into and widening the cervix (Figure 1D). Additionally, bilateral ovarian cysts were also observed (Figures 1E-1F).

Given these findings, the patient was planned for a total abdominal hysterectomy with bilateral salpingo-oophorectomy (TAH & BSO). However, intraoperatively, the staging laparotomy procedure was converted to a modified radical hysterectomy with infracolic omentectomy and bilateral pelvic lymph node dissection under general and epidural anesthesia. During the surgery, an omental mass was identified and sent for HPE analysis.

**Figure 2:**
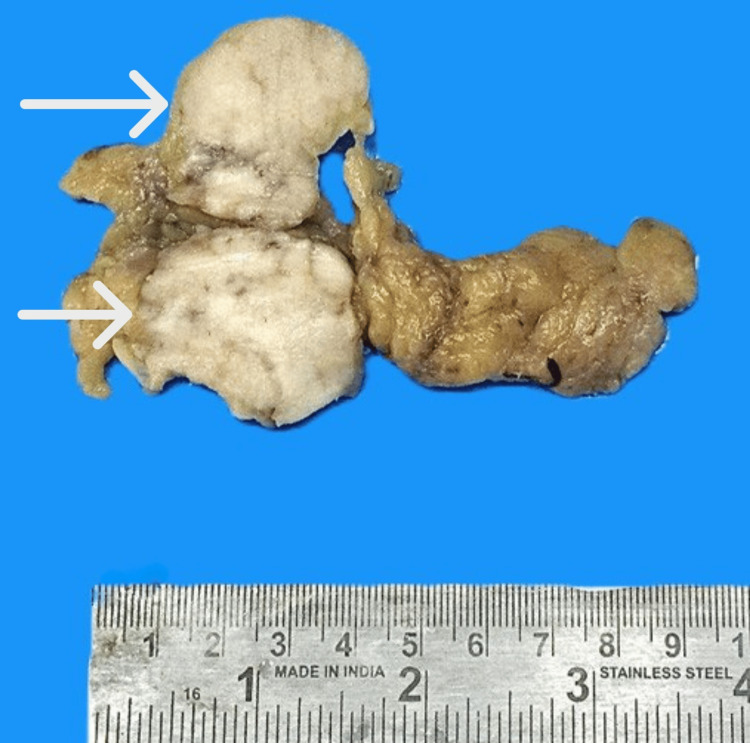
Infracolic omentectomy specimen showing nodular growth (white arrows)

A gross examination of the omental mass specimen showed nodular growth along with attached fatty tissue, measuring 10.5 x 4 x 2 cm. The nodular mass measured 4.5 x 3.5 x 2 cm. The cut surface of the nodule was grey-white to grey-brown, homogeneous, and firm (Figure 2).

**Figure 3:**
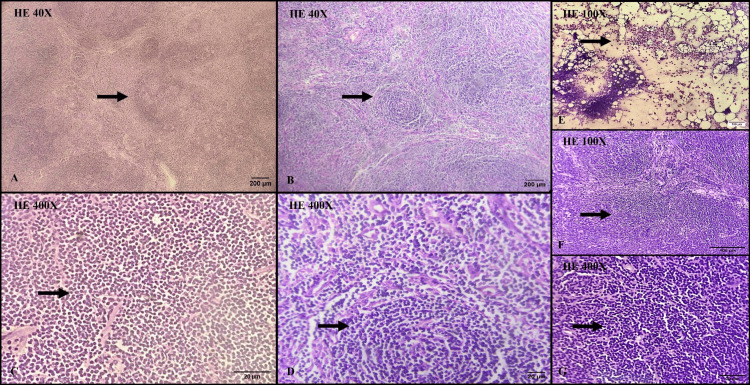
Microscopic examination of the nodular mass in scanner view (H&E, 40X magnification) showed effacement of nodal architecture with sheets of lymphoid cells (arrows in A and B). Microscopic examination of the nodular mass in high-power view (H&E, 400X magnification) revealed a monomorphous population of small, cleaved lymphocytes with round nuclei, dense chromatin, and scant cytoplasm (arrows in C and D). Microscopic examination of bone marrow aspiration showed normocellular marrow with trilineage hematopoiesis (arrows in E). Microscopic examination of lymph nodes showed involvement by neoplastic lymphocytes (arrows in F and G) H&E: hematoxylin and eosin

Microscopic examination of the nodular lesion revealed sheets of lymphoid cells arranged in closely packed, variably sized follicles (Figures 3A-3B). Some follicles are well-defined, while others appear ill-defined and are composed of a monomorphic population of small, cleaved lymphocytes with scant cytoplasm, round nuclei (centrocytes), and dense chromatin. These follicles lack both germinal centers and mantle zones, containing a scant number of centroblasts. Tingible body macrophages are absent (Figures 3C-3D). 

Bone marrow examination revealed no involvement of NHL cells and displayed a normocellular marrow with trilineage hematopoiesis (Figure 3E). The bilateral pelvic lymph nodes submitted revealed involvement by NHL cells (Figures 3F-3G).

**Figure 4:**
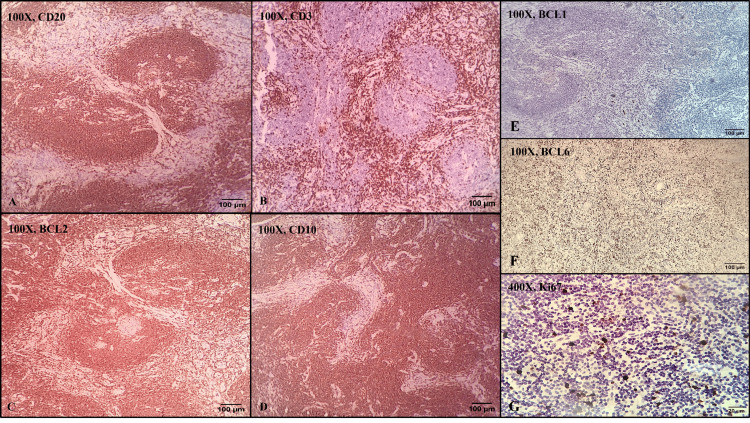
Immunohistochemical examination: A: CD20 showed strong membranous positivity in the neoplastic lymphocytes (100X magnification); B: CD3 showed strong cytoplasmic and membranous positivity in the interspersed lymphocytes (100X magnification); C: BCL2 showed strong membranous and cytoplasmic positivity in the neoplastic lymphocytes (100X magnification); D: CD10 showed strong membranous positivity in the neoplastic lymphocytes (100X magnification); E: BCL1 negative in the neoplastic lymphocytes (100X magnification); F: BCL6 showed strong nuclear positivity in the neoplastic lymphocytes (100X magnification); G: Ki67 showed nuclear positivity in 8-10% of neoplastic lymphocytes (400X magnification) CD: cluster of differentiation; BCL: B-cell lymphoma

All the HPE findings were suggestive of small-cell NHL. Immunohistochemistry (IHC) was performed for further classifying NHL using the cluster of differentiation 3 (CD3), CD20, CD10, and B-cell lymphoma 2 (BCL2). CD20 showed strong membranous positivity in neoplastic lymphocytes within the poorly formed germinal center areas (Figure 4A). CD3 showed strong membranous and cytoplasmic positivity in the interspersed T-lymphocytes within the parafollicular region (Figure 4B). BCL2 showed diffusely strong cytoplasmic and membranous positivity in the neoplastic lymphocytes within the germinal center tumor cells (Figure 4C). CD10 showed strong membranous positivity in neoplastic lymphocytes within the poorly formed germinal center areas (Figure 4D). To rule out MCL from FL, BCL1 (cyclin D1) IHC was performed, which showed no staining in the neoplastic lymphocytes (Figure 4E). To rule out MZL from FL, BCL6 IHC was performed, which showed strong nuclear positivity in neoplastic lymphocytes (Figure 4F). Ki67 IHC demonstrated 8% to 10% nuclear positivity in the neoplastic lymphocytes, indicating a low proliferative index (Figure 4G). Based on the HPE and IHC findings, a final diagnosis of FL, grade 1, was made, and the disease was classified as stage II, given the involvement of the omentum and pelvic lymph nodes without bone marrow infiltration.

In our case, in terms of treatment, the patient underwent complete surgical removal of the tumor followed by HPE and IHC examination. A post-operative PET-CT scan conducted at an outside diagnostic center revealed no abnormal metabolic activity, indicating the absence of residual disease. Although adjuvant chemotherapy was recommended to minimize the risk of recurrence, the patient opted not to pursue further treatment. The patient is doing well at her two-month postoperative follow-up.

## Discussion

FL is a slowly progressing type of NHL, which has a more favorable prognosis than other lymphomas [16]. It originates from B cells in the germinal centers of lymph nodes, bone marrow, or spleen. Understanding FL involves evaluating its symptoms, diagnosis, molecular basis, and treatment [16].

The majority of individuals with FL show no symptoms, but symptoms can include fever, weight loss, night sweats, fatigue, or recurrent infections. Extranodal FL, though rare, may cause location-specific symptoms. For instance, bone marrow involvement leads to anemia in about 10% of cases, while leukopenia and thrombocytopenia are uncommon [17,18].

Primary mesenteric tumors are rare, occurring in fewer than 1 in 200,000, with FL being the most prevalent type [12,13]. Given its slow progression, FL is often discovered incidentally during imaging studies for unrelated health issues or during investigations for other medical conditions [19].

Diagnosing FL involves histopathology, immunophenotyping, and genetic analysis. Histologically, FL shows follicle center cell proliferation in a nodular pattern. IHC typically detects markers like CD10, CD19, BCL6, and BCL2, with CD5 being absent [18]. The key genetic feature is the t(14;18) translocation, which links the BCL2 gene to the heavy chain immunoglobulin locus, leading to the overproduction of BCL2 protein and inhibition of apoptosis [20].

The management of FL usually involves a comprehensive, multidisciplinary approach that includes surgical intervention, chemotherapy, and immunotherapy [21]. 

Despite the uncommon presentation of FL in the omentum, the overall prognosis remains generally positive, especially if the disease is detected early and managed effectively [12,13]. However, the rare presentation in the omentum may suggest more complex disease behavior or a heightened risk of spread, necessitating vigilant monitoring for potential recurrence or transformation.

## Conclusions

This case of FL presenting in an unusual site, such as the omentum, underscores the critical importance of recognizing and understanding atypical presentations of FL, particularly in rare and extranodal locations. The omental involvement in this case, as a primary solitary nodular mass, represents an uncommon manifestation; however, it adhered to the typical HPE and immunohistochemical profile of FL. This reinforces the need for thorough and comprehensive diagnostic evaluations in such unusual cases. The accurate diagnosis and detailed pathological assessment of this patient highlight the vital role of histopathology in guiding prognosis and formulating effective treatment strategies for FL, especially in cases with non-traditional presentations. This case not only broadens our understanding of the clinical spectrum of FL but also emphasizes the necessity for continued research and vigilance in managing rare and extranodal presentations of lymphoma, ensuring that such atypical cases are effectively recognized and treated.

## References

[1] Jaffe ES, Harris NL, Swerdlow SH (2017). Follicular lymphoma. WHO Classification of Tumours of Haematopoietic and Lymphoid Tissues.

[2] Swerdlow SH, Campo E, Pileri SA (2016). The 2016 revision of the World Health Organization classification of lymphoid neoplasms. Blood.

[3] Armitage JO, Weisenburger DD (1998). New approach to classifying non-Hodgkin’s lymphomas: clinical features of the major histologic subtypes. Non-Hodgkin’s lymphoma classification project. J Clin Oncol.

[4] Sylvia MT, Dey B, Basu D, Jacob SE, Kar R, Dubashi B (2016). Follicular lymphoma: a clinicopathological analysis from a tertiary care Institute in southern India. Mediterr J Hematol Infect Dis.

[5] (1997). A clinical evaluation of the International lymphoma Study Group classification of non-Hodgkin’s lymphoma. The non-Hodgkin’s lymphoma classification Project. Blood.

[6] Kurz KS, Kalmbach S, Ott M, Staiger AM, Ott G, Horn H (2023). Follicular lymphoma in the fifth edition of the WHO classification of haematolymphoid neoplasms - updated classification and new biological data. Cancers (Basel).

[7] Otter R, Gerrits WB (1989). Primary extranodal and nodal non-Hodgkin’s lymphoma. A survey of a population-based registry. Eur J Cancer Clin Oncol.

[8] Psyrri A, Papageorgiou S, Economopoulos T (2008). Primary extranodal lymphomas of stomach: clinical presentation, diagnostic pitfalls and management. Ann Oncol.

[9] Herrmann R, Panahon AM, Barcos MP (1980). Gastrointestinal involvement in non-Hodgkin's lymphoma. Cancer.

[10] d'Amore F, Brincker H, Grønbaek K (1994). Non-Hodgkin's lymphoma of the gastrointestinal tract: a population-based analysis of incidence, geographic distribution, clinicopathologic presentation features, and prognosis. Danish Lymphoma Study Group. J Clin Oncol.

[11] Groves FD, Linet MS, Travis LB, Devesa SS (2000). Cancer surveillance series: non-Hodgkin's lymphoma incidence by histologic subtype in the United States from 1978 through 1995. J Natl Cancer Inst.

[12] Devesa SS, Fears T (1992). Non-Hodgkin's lymphoma time trends: United States and international data. Cancer Res.

[13] Nishimura Y, Yamamoto A, Takahara M, Otsuka F (2019). Mesenteric follicular lymphoma. Clin Case Rep.

[14] Sheth S, Horton KM, Garland MR, Fishman EK (2003). Mesenteric neoplasms: CT appearances of primary and secondary tumors and differential diagnosis. Radiographics.

[15] Yoo E, Kim JH, Kim MJ, Yu JS, Chung JJ, Yoo HS, Kim KW (2007). Greater and lesser omenta: normal anatomy and pathologic processes. Radiographics.

[16] Freedman A (2018). Follicular lymphoma: 2018 update on diagnosis and management. Am J Hematol.

[17] Sarkozy C, Baseggio L, Feugier P (2014). Peripheral blood involvement in patients with follicular lymphoma: a rare disease manifestation associated with poor prognosis. Br J Haematol.

[18] Bain BJ (2018). Diagnosis of follicular lymphoma from the peripheral blood. Am J Hematol.

[19] West RB, Warnke RA, Natkunam Y (2002). The usefulness of immunohistochemistry in the diagnosis of follicular lymphoma in bone marrow biopsy specimens. Am J Clin Pathol.

[20] Cleary ML, Sklar J (1985). Nucleotide sequence of a t(14;18) chromosomal breakpoint in follicular lymphoma and demonstration of a breakpoint-cluster region near a transcriptionally active locus on chromosome 18. Proc Natl Acad Sci U S A.

[21] Jacobsen E (2022). Follicular lymphoma: 2023 update on diagnosis and management. Am J Hematol.

